# Supplementary material for: The effect of host community functional traits on plant disease risk varies along an elevational gradient
Source: eLife. 2021 May 13;10:e67340. doi: 10.7554/eLife.67340 (PMC8208817; doi:10.7554/eLife.67340)
Supplement: Supplementary file 1. — (a) Timing of grazing, vegetation and disease surveys and temperature measurements at each site. Recovery days represents the amount of time between the end of grazing activities and the beginning of the vegetation survey at each site. (b) Results of Type II Analysis of Deviance tests on models quantifying the effects of soil moisture and either soil-surface temperature, soil temperature, air temperature, or elevation on two measures of host community structure (Host Richness, Host Pace-of-Life). (c) Results of Type II Analysis of Deviance test on mixed models of disease, using soil temperature, air temperature, or elevation to evaluate factors that influenced square-root transformed community parasite load. (d) Coefficient estimates from the structural equation model fit with mean-centered soil-surface temperature and host pace-of-life. Estimates are provided both raw (Estimate) and scaled by the ratio of the standard deviation of x divided by the standard deviation of y (Std Estimate) to facilitate comparisons. Correlations among dependent variables are indicated by ~~. (e) Calanda Biodiversity Observatory Vegetation list. This list includes species that were observed during the vegetation survey as well as taxa observed outside of the plots during extensive preliminary surveys of Mount Calanda. (f) Comparison of different models quantifying the relationship between host community traits and disease. Each model contained square-root transformed community parasite load as the response, and elevation, host community species richness, richness-independent phylogenetic diversity, and some combination of host traits as fixed effects. To estimate whether the effect of host community structure depends on elevation, we also included in the model the pairwise interactions between each measure of host community structure and elevation as additional fixed effects, The Pace-of-Life model includes host community pace-of-life as a latent factor, and is the model reported in the [file elife-67340-supp1.docx]

**Supplementary File 1**

**Supplementary File 1a.**

| **Site** | **Meadow** | **Elevation** | **Date of the vegetation survey** | **Recovery days** | **Date of the disease survey** | **Date of the temperature measurements** |
| --- | --- | --- | --- | --- | --- | --- |
| I1 | Im Bofel | 766.3m | 10.7.2019 | 24 | 15.8.2019 | 7.8.­–9.9.2019 |
| I2 | Im Bofel | 737.4m | 4.7.2019 | 18 | 13.–15.8.2019 | 7.8.–9.9.2019 |
| I3 | Im Bofel | 711.5m | 3.–4.7.2019 | 17–18 | 29.7.2019 | 7.–28.8.2019 |
| I4 | Im Bofel | 702.4m | 2.–3.7.2019 | 16–17 | 29.–30.7.2019 | 7.–28.8.2019 |
| I5 | Im Bofel | 684.5m | 27.6.2019–2.7.2019 | 11–16 | 18.–30.7.2019 | 7.–28.8.2019 |
| I6 | Im Bofel | 702.2m | 28.6.2019 | 12 | 13.8.2019 | 7.8.–11.9.2019 |
| I7 | Im Bofel | 648.5m | 5.–9.7.2019 | 19–23 | 30.7.2019 | 7.8.–11.9.2019 |
| A1 | Arella | 1020.6m | 16.–17.7.2019 | 17–18 | 8.8.2019 | 7.8.–12.9.2019 |
| A2 | Arella | 984.9m | 16.7.2019 | 17 | 15.–19.8.2019 | 7.8.–12.9.2019 |
| A3 | Arella | 949.8m | 15.–16.7.2019 | 16–17 | 8.8.2019 | 7.8.–11.9.2019 |
| A4 | Arella | 1002.8m | 12.7.2019 | 13 | 22.8.2019 | 7.8.–11.9.2019 |
| A5 | Arella | 1001m | 11.7.2019 | 12 | 7.8.2019 | 7.8.–11.9.2019 |
| A6 | Arella | 981.9m | 10.–11.7.2019 | 11–12 | 7.8.2019 | 7.8.–11.9.2019 |
| N1 | Nesselboden | 1390.3m | 23.7.2019 | 20 | 14.8.2019 | 7.8.–12.9.2019 |
| N2 | Nesselboden | 1405.5m | 22.–23.7.2019 | 19–20 | 2.8.2019 | 7.8.–12.9.2019* |
| N3 | Nesselboden | 1407.7m | 19.–22.7.2019 | 16–19 | 2.–6.8.2019 | 7.8.–12.9.2019* |
| N4 | Nesselboden | 1420m | 19.7.2019 | 16 | 6.–14.8.2019 | 7.8.–12.9.2019* |
| N5 | Nesselboden | 1398.6m | 17.–19.7.2019 | 14–16 | 19.8.2019 | 7.8.–12.9.2019* |
| O3 | Oberberg – Under Alp | 1612.7m | 24.7.2019 | 5 | 31.7.2019 | 7.8.–8.9.2019 |
| O4 | Oberberg – Under Alp | 1576.2m | 23.–24.7.2019 | 4–5 | 31.7.2019 | 7.8.–9.9.2019 |
| U1 | Oberberg – Under Alp | 1745.8m | 25.7.–1.8.2019 | 6–13 | 16.8.2019 | 7.8.–8.9.2019 |
| U2 | Oberberg – Under Alp | 1749.2m | 26.7.–1.8.2019 | 7–13 | 16.8.2019 | 7.8.–8.9.2019 |
| *Data loggers were removed for 12 days 30.8.–10.9.2019 due to escape of cattle from higher elevation meadows. | | | | | | |

**Supplementary File 1b.**

|  | | *Host Richness response* | | | |  | *Host Pace-of-Life response* | | | |
| --- | --- | --- | --- | --- | --- | --- | --- | --- | --- | --- |
| **Predictor** | | **Estimate** | **Chisq** | **Df** | **P** |  | **Estimate** | **Chisq** | **Df** | **P** |
|  | *Model using Soil-surface Temperature* | | | | | | | | | |
| Temperature | | -0.8000 | 7.046 | 1 | 0.008 |  | 0.0559 | 6.564 | 1 | 0.010 |
| Soil Moisture | | -3.0576 | 0.019 | 1 | 0.891 |  | 1.9910 | 2.193 | 1 | 0.139 |
|  | *Model using Soil Temperature* | | | | | | | | | |
| Temperature | | -0.5583 | 2.027 | 1 | 0.155 |  | 0.0610 | 6.605 | 1 | 0.010 |
| Soil Moisture | | -10.7442 | 0.201 | 1 | 0.654 |  | 2.4685 | 3.443 | 1 | 0.064 |
|  | *Model using Air Temperature* | | | | | | | | | |
| Temperature | | -0.8140 | 9.352 | 1 | 0.002 |  | 0.0560 | 6.069 | 1 | 0.014 |
| Soil Moisture | | -4.0575 | 0.037 | 1 | 0.848 |  | 2.1489 | 2.619 | 1 | 0.106 |
|  | *Model using Elevation* | | | | | | | | | |
| Elevation | | 0.0032 | 4.106 | 1 | 0.043 |  | -0.0002 | 3.385 | 1 | 0.066 |
| Soil Moisture | | -10.3180 | 0.204 | 1 | 0.652 |  | 2.5128 | 3.133 | 1 | 0.077 |

**Supplementary File 1c.**

| **Predictor** | **Estimate** | **Chisq** | **Df** | **P** |
| --- | --- | --- | --- | --- |
| *Model using soil temperature* |  |  |  |  |
| Soil Temperature | 0.0521 | 9.2048 | 1 | 0.002 |
| Soil Moisture | 0.1401 | 0.0236 | 1 | 0.878 |
| Host Richness | -0.0098 | 6.9059 | 1 | 0.009 |
| Host Pace-of-Life | 0.1240 | 1.2631 | 1 | 0.261 |
| Temperature $\times$ Richness | 0.0050 | 3.6899 | 1 | 0.055 |
| Temperature $\times$ Pace-of-Life | 0.1456 | 14.8832 | 1 | <0.001 |
| Moisture $\times$ Richness | -0.2688 | 2.5686 | 1 | 0.109 |
| Moisture $\times$ Pace-of-Life | -0.9975 | 0.1405 | 1 | 0.708 |
| *Model using air temperature* |  |  |  |  |
| Air Temperature | 0.0416 | 7.2553 | 1 | 0.007 |
| Soil Moisture | -0.1179 | 0.0354 | 1 | 0.851 |
| Host Richness | -0.0080 | 4.6625 | 1 | 0.031 |
| Host Pace-of-Life | 0.1437 | 1.9957 | 1 | 0.158 |
| Temperature $\times$ Richness | 0.0039 | 3.1190 | 1 | 0.077 |
| Temperature $\times$ Pace-of-Life | 0.1187 | 11.6772 | 1 | <0.001 |
| Moisture $\times$ Richness | -0.2804 | 2.6645 | 1 | 0.103 |
| Moisture $\times$ Pace-of-Life | -1.7399 | 0.4140 | 1 | 0.520 |
| *Model using elevation* |  |  |  |  |
| Elevation | -0.0002 | 6.4167 | 1 | 0.011 |
| Soil Moisture | -0.0294 | 0.0025 | 1 | 0.960 |
| Host Richness | -0.0099 | 6.0337 | 1 | 0.014 |
| Host Pace-of-Life | 0.1371 | 2.5213 | 1 | 0.112 |
| Elevation$\times$ Richness | -0.00002 | 1.9509 | 1 | 0.162 |
| Elevation$\times$ Pace-of-Life | -0.0006 | 12.9235 | 1 | <0.001 |
| Moisture $\times$ Richness | -0.2923 | 2.9168 | 1 | 0.088 |
| Moisture $\times$ Pace-of-Life | -1.2409 | 0.2130 | 1 | 0.644 |

**Supplementary File 1d.**

| **Response** | **Predictor** | **Estimate** | **Std Error** | **DF** | **Critical Value** | **P** | **Std Estimate** |
| --- | --- | --- | --- | --- | --- | --- | --- |
| Square-root transformed community parasite load | Soil-surface Temperature | 0.0446 | 0.0128 | 16 | 3.4861 | 0.0031 | 0.2430 |
|  | Host Species Richness | -0.0102 | 0.0039 | 107 | -2.5891 | 0.0110 | -0.1489 |
|  | Host Pace-of-Life | 0.1289 | 0.0681 | 107 | 1.8933 | 0.0610 | 0.1149 |
|  | Temperature × Pace-of-Life | 0.1023 | 0.0341 | 107 | 2.9998 | 0.0034 | 0.1797 |
|  |  |  |  |  |  |  |  |
| Host Species Richness | Soil-surface Temperature | -0.8069 | 0.2871 | 16 | -2.8103 | 0.0126 | -0.2996 |
| Host Pace-of-Life | Soil-surface Temperature | 0.0641 | 0.0232 | 16 | 2.7623 | 0.0139 | 0.3919 |
|  |  |  |  |  |  |  |  |
| Soil-surface Temperature | Elevation | -0.0041 | 0.0004 | 23 | -11.532 | 0.0000 | -0.9126 |
|  |  |  |  |  |  |  |  |
| ~~ Host Species Richness | ~~ Host Pace-of-Life | -0.0848 | NA | 220 | -1.2542 | 0.1056 | -0.0848 |
| Goodness of fit: Fisher’s C = 4.111 with p = 0.662 and on 6 degrees of freedom | | | | | | | |

**Supplementary File 1e.**

| **Species** | **Habitat preference (Flora Helvetica)** |
| --- | --- |
| *Achillea millefolium* | collin-subalpin (-alpin) |
| *Acinos alpinus* | (montane) subalpine (-alpine) |
| *Acinos arvensis* | kollin-montan (-subalpin) |
| *Aconitum lycoctonum* | kollin-subalpin |
| *Aconitum napellus* | subalpine-alpine |
| *Aegopodium podagraria* | kollin-montan (-subalpin) |
| *Agrimonia eupatoria* | kollin-montan (-subalpin) |
| *Agrostis alpina* | subalpin-alpin |
| *Agrostis capillaris* | kollin-subalpin (-alpin) |
| *Agrostis gigantea* | kollin-subalpin |
| *Agrostis stolonifera* | kollin-subalpin (-alpin) |
| *Agrostis schraderiana* | (montane) subalpine-alpine |
| *Ajuga pyramidalis* | (montane) subalpine-alpine |
| *Ajuga reptans* | kollin-subalpin (-alpin) |
| *Alchemilla conjuncta* | montane-subalpin (-alpin) |
| *Alchemilla xanthochlora* | kollin-alpin |
| *Alchemilla pratensis* | kollin-alpin |
| *Alchemilla nitida* | subalpine (-alpin) |
| *Allium carinatum* | kollin-subalpin |
| *Anacamptis pyramidalis* | Kollin-montan |
| *Androsace chamaejasme* | (montane) subalpine-alpine |
| *Androsace obtusifolia* | (subalpine) alpine |
| *Anemone narcissiflora* | (montane) subalpine (-alpine) |
| *Anthericum ramosum* | kollin-subalpin (-alpin) |
| *Anthoxanthum alpinum* | (montane) subalpine-alpine |
| *Anthoxanthum odoratum* | kollin-alpin |
| *Anthriscus sylvestris* | kollin-subalpin |
| *Anthyllis vulneraria* | kollin-montan (-subalpin) |
| *Aquilegia atrata* | (kollin-) montan-subalpin |
| *Arabis ciliata* | (collin-) subalpine (-alpin) |
| *Arenaria ciliata* | (subalpine) alpine |
| *Arenaria serpyllifolia* | Kollin-montan (-subalpin) |
| *Arnica montana* | (montane) subalpine-alpine |
| *Arrhenatherum elatius* | kollin-montan (-subalpin) |
| *Asperula cynanchica* | kollin-montan (-subalpin) |
| *Aster alpinus* | subalpin-alpin |
| *Aster amellus* | kollin-montan |
| *Aster bellidiastrum* | (kollin-) montan-alpin |
| *Astragalus glycyphyllos* | kollin-montan (-subalpin) |
| *Avenula pubescens* | kollin-subalpin (-alpin) |
| *Bellis perennis* | collin-subalpin (-alpin) |
| *Berberis vulgaris* | kollin-subalpin |
| *Bothriochloa ischaemum* | kollin-montan |
| *Botrychium lunaria* | (kollin-) montan-alpin |
| *Brachypodium pinnatum* | kollin-subalpin |
| *Briza media* | kollin-subalpin (-alpin) |
| *Bromus erectus* | kollin-montan (-subalpin) |
| *Bromus hordeaceus* | kollin-montan (-subalpin) |
| *Bromus inermis* | kollin-montan (-subalpin) |
| *Buphthalmum salicifolium* | kollin-subalpin |
| *Calystegia sepium* | kollin-montan |
| *Campanula cochleariifolia* | (kollin-) montan-alpin |
| *Campanula glomerata* | Kollin-montan (-subalpin) |
| *Campanula patula* | kollin-montan (-subalpin) |
| *Campanula rapunculoides* | kollin-montan (-subalpin) |
| *Campanula rotundifolia* | kollin-subalpin (-alpin) |
| *Campanula scheuchzeri* | (montan-) subalpine-alpine |
| *Carduus defloratus* | montan-alpin |
| *Carduus nutans* | kollin-subalpin |
| *Carex capillaris* | (montane) subalpine-alpine |
| *Carex caryophyllea* | kollin-subalpin (-alpin) |
| *Carex flacca* | kollin-subalpin (-alpin) |
| *Carex montana* | kollin-subalpin |
| *Carex ornithopoda* | kollin-subalpin (-alpin) |
| *Carex parviflora* | subalpine-alpine |
| *Carex sempervirens* | (montane) subalpine-alpine |
| *Carlina acaulis* | (collinous) montane-subalpin |
| *Carlina vulgaris* | Kollin-montan |
| *Carum carvi* | kollin-subalpin (-alpin) |
| *Centaurea jacea* | kollin-subalpin |
| *Centaurea montana* | montane-subalpine |
| *Centaurea scabiosa* | kollin-montan (-subalpin) |
| *Centaurium erythraea* | kollin-montan |
| *Cephalanthera longifolia* | kollin-montan |
| *Cerastium alpinum* | (subalpine) alpine |
| *Cerastium fontanum* | kollin-montan (-subalpin) |
| *Chenopodium album* | kollin-subalpin |
| *Chenopodium bonus-henricus* | (collin-) montan-subalpin (-alpin) |
| *Cichorium intybus* | kollin-montan (-subalpin) |
| *Cirsium acaule* | (collin) montane-subalpin (-alpin) |
| *Cirsium arvense* | kollin-montan (-subalpin) |
| *Cirsium spinosissimum* | subalpine-alpine |
| *Clinopodium vulgare* | kollin-subalpin (-alpin) |
| *Colchicum autumnale* | kollin-subalpin |
| *Conyza canadensis* | kollin-montan (-subalpin) |
| *Crepis biennis* | Kollin-montan (-subalpin) |
| *Crocus albiflorus* | montan-alpin |
| *Cynosurus cristatus* | kollin-subalpin |
| *Dactylis glomerata* | kollin-subalpin (-alpin) |
| *Danthonia decumbens* | (kollin-) montane-subalpine |
| *Daucus carota* | kollin-montan (-subalpin) |
| *Deschampsia cespitosa* | kollin-alpin |
| *Dianthus superbus* | (collin) subalpin (-alpin) |
| *Dianthus sylvestris* | kollin-subalpin (-alpin) |
| *Digitaria sanguinalis* | kollin-montan |
| *Dryas octopetala* | subalpin-alpin |
| *Echinochloa crus-galli* | kollin-montan |
| *Echium vulgare* | kollin-subalpin |
| *Elyna myosuroides* | subalpine-alpine |
| *Erica carnea* | (kollin-) montane-subalpine (-alpin) |
| *Erigeron neglectus* | (subalpine) alpine |
| *Erodium cicutarium* | kollin-montan (-subalpin) |
| *Euphorbia cyparissias* | kollin-alpin |
| *Euphrasia minima* | subalpine-alpine |
| *Euphrasia rostkoviana montana* | kollin-alpin |
| *Euphrasia salisburgensis* | kollin-alpin |
| *Festuca arundinacea* | kollin-montan (-subalpin) |
| *Festuca ovina* | kollin-alpin |
| *Festuca quadriflora* | subalpine-alpine |
| *Festuca rubra* | kollin-alpin |
| *Filipendula ulmaria* | kollin-subalpin |
| *Fragaria vesca* | kollin-subalpin |
| *Gagea fragifera* | subalpin-alpin |
| *Galium mollugo* | kollin-montan |
| *Galium verum* | kollin-montan (-subalpin) |
| *Gentiana acaulis* | (montane) subalpine-alpine |
| *Gentiana brachyphylla* | alpine |
| *Gentiana campestris* | montan-subalpin (-alpin) |
| *Gentiana clusii* | (montane) subalpine-alpine |
| *Gentiana verna* | montane-alpine |
| *Geranium columbinum* | kollin-montan |
| *Geranium pyrenaicum* | kollin-montan (-subalpin) |
| *Geranium robertianum* | kollin-montan (-subalpin) |
| *Geranium sylvaticum* | (collinous) montane-subalpin (-alpin) |
| *Geum montanum* | (montane) subalpine-alpine |
| *Globularia nudicaulis* | (montane) subalpine (-alpine) |
| *Gymnadenia conopsea* | kollin-subalpin (-alpin) |
| *Helianthemum alpestre* | (montane) subalpine-alpine |
| *Helianthemum nummularium* | kollin-alpin |
| *Helictotrichon pubescens* | kollin-subalpin (-alpin) |
| *Helictotrichon versicolor* | (montane) subalpine-alpine |
| *Helictotrichon pubescens* | kollin-subalpin (-alpin) |
| *Hepatica nobilis* | kollin-montan (-subalpin) |
| *Hieracium lactucella* | kollin-subalpin |
| *Hieracium pilosella* | kollin-alpin |
| *Hippocrepis comosa* | kollin-subalpin (-alpin) |
| *Homogyne alpina* | (montane) subalpine-alpine |
| *Hordeum murinum* | kollin-montan |
| *Hypericum maculatum* | Montan-alpin |
| *Hypericum perforatum* | kollin-montan (-subalpin) |
| *Knautia arvensis* | kollin-montan (-subalpin) |
| *Koeleria pyramidata* | kollin-subalpin |
| *Lamium maculatum* | kollin-subalpin (-alpin) |
| *Larix decidua* | subalpine |
| *Laserpitium siler* | (kollin-) montan-subalpin |
| *Lathyrus niger* | kollin (-montan) |
| *Lathyrus pratensis* | kollin-montan |
| *Lathyrus vernus* | kollin-montan |
| *Leontodon hispidus* | kollin-alpin |
| *Leucanthemum vulgare* | kollin-subalpin (-alpin) |
| *Ligustrum vulgare* | kollin (-montan) |
| *Linum catharticum* | kollin-subalpin |
| *Lithospermum arvense* | kollin-montan (-subalpin) |
| *Lolium perenne* | kollin-montan (-subalpin) |
| *Lotus corniculatus* | kollin-subalpin (-alpin) |
| *Lotus maritimus* | collin-montan (-subalpin) |
| *Luzula campestris* | kollin-montan (-subalpin) |
| *Luzula nivea* | (collinous) montane-subalpine |
| *Luzula luzuloides* | kollin-montan (-subalpin) |
| *Luzula sudetica* | montan-subalpin |
| *Lysimachia nummularia* | Kollin-montan (-subalpin) |
| *Malva neglecta* | kollin-montan (-subalpin) |
| *Medicago falcata* | kollin-subalpin |
| *Medicago lupulina* | kollin-montan (-subalpin) |
| *Medicago minima* | kollin-montan |
| *Medicago sativa* | kollin-montan (-subalpin) |
| *Melampyrum pratense* | kollin-subalpin (-alpin) |
| *Molinia caerulea* | kollin-subalpin (-alpin) |
| *Myosotis alpestris* | (montan-) subalpine-alpine |
| *Nardus stricta* | montane-alpine |
| *Odontites luteus* | kollin-montan (-subalpin) |
| *Onobrychis sp* | kollin-montan (-subalpin) |
| *Ononis spinosa* | kollin-montan |
| *Ophrys sphegodes* | kollin (-montan) |
| *Orchis mascula* | kollin-subalpin (-alpin) |
| *Orchis militaris* | kollin-montan |
| *Orchis ustulata* | kollin-subalpin |
| *Origanum vulgare* | kollin-subalpin |
| *Parnassia palustris* | kollin-alpin |
| *Pastinaca sativa* | kollin-montan (-subalpin) |
| *Peucedanum oreoselinum* | kollin-montan (-subalpin) |
| *Phleum pratense* | collin-montan (-subalpin) |
| *Phyteuma hemisphaericum* | (subalpine) alpine |
| *Phyteuma orbiculare* | montane-subalpine (-alpine) |
| *Picea abies* | (collinous) montane-subalpine |
| *Pimpinella saxifraga* | kollin-subalpin |
| *Pilosella lactucella* | kollin-subalpin |
| *Pilosella officinarum* | kollin-alpin |
| *Plantago alpina* | (montane) subalpine-alpine |
| *Plantago atrata* | (montane) subalpine-alpine |
| *Plantago lanceolata* | collin-subalpin (-alpin) |
| *Plantago major* | kollin-subalpin (-alpin) |
| *Plantago media* | kollin-montan (-subalpin) |
| *Platanthera chlorantha* | kollin-subalpin |
| *Poa alpina* | (collinous) subalpine-alpine |
| *Poa annua* | kollin-subalpin (-alpin) |
| *Poa badensis* | kollin-montan |
| *Poa pratensis* | collin-subalpin (-alpin) |
| *Polygala alpestris* | (montane) subalpine (-alpine) |
| *Polygala chamaebuxus* | (collinous) montane-subalpine (-alpin) |
| *Polygala comosa* | kollin-subalpin |
| *Polygala vulgaris* | kollin-montan (-subalpin) |
| *Polygonatum odoratum* | kollin-subalpin |
| *Polygonum aviculare* | kollin-montan (-subalpin) |
| *Polygonum viviparum* | (montane) subalpine-alpine |
| *Potentilla anserina* | kollin-montan (-subalpin) |
| *Potentilla aurea* | (montane) subalpine-alpine |
| *Potentilla crantzii* | (montane) subalpine-alpine |
| *Potentilla erecta* | kollin-subalpin (-alpin) |
| *Primula auricula* | (collin) subalpine-alpine |
| *Primula elatior* | kollin-subalpin (-alpin) |
| *Primula farinosa* | (kollin-) montan-alpin |
| *Primula integrifolia* | (subalpine) alpine |
| *Primula veris* | Kollin-Subalpin |
| *Prunella grandiflora* | kollin-subalpin (-alpin) |
| *Prunella vulgaris* | kollin-subalpin (-alpin) |
| *Pteridium aquilinum* | kollin-subalpin |
| *Pulsatilla alpina subsp. alpina* | subalpine-alpine |
| *Pulsatilla vernalis* | (montane) subalpine-alpine |
| *Anemone pulsatilla* | kollin-montan |
| *Ranunculus repens* | kollin-subalpin |
| *Ranunculus acris* | kollin-subalpin (-alpin) |
| *Ranunculus alpestris* | (montane) subalpine-alpine |
| *Ranunculus bulbosus* | kollin-subalpin |
| *Ranunculus montanus* | (montane) subalpine-alpine |
| *Ranunculus tuberosus* | kollin-subalpin |
| *Reseda lutea* | kollin-montan (-subalpin) |
| *Rhinanthus alectorolophus* | kollin-subalpin (-alpin) |
| *Rubus fruticosus* | kollin-subalpin |
| *Rubus idaeus* | collin-subalpin (-alpin) |
| *Rubus saxatilis* | collin-subalpin (-alpin) |
| *Rumex acetosella* | kollin-subalpin |
| *Rumex alpestris* | montane-subalpine (-alpine) |
| *Rumex alpinus* | (montane) subalpine (-alpine) |
| *Salvia pratensis* | kollin-montan (-subalpin) |
| *Sanguisorba minor* | kollin-subalpin |
| *Saponaria ocymoides* | kollin-subalpin |
| *Scabiosa columbaria* | Kollin-Montan |
| *Scabiosa lucida* | (montane) subalpine-alpine |
| *Sedum album* | kollin-subalpin (-alpin) |
| *Selaginella selaginoides* | (montane) subalpine-alpine |
| *Senecio alpinus* | subalpin-alpin |
| *Seseli annuum* | kollin-montan |
| *Sesleria caerulea* | (kollin-) montan-alpin |
| *Setaria viridis* | kollin-montan |
| *Silene nutans* | kollin-subalpin (-alpin) |
| *Silene vulgaris* | kollin-subalpin (-alpin) |
| *Solanum dulcamara* | kollin-montan (-subalpin) |
| *Soldanella alpina* | (montane) subalpine-alpine |
| *Stachys officinalis* | kollin-montan |
| *Stachys recta* | kollin-montan (-subalpin) |
| *Stellaria media* | kollin-subalpin |
| *Taraxacum officinale* | kollin-subalpin (-alpin) |
| *Taraxacum campylodes* | kollin-subalpin (-alpin) |
| *Teucrium chamaedrys* | kollin-subalpin |
| *Teucrium montanum* | kollin-subalpin (-alpin) |
| *Thalictrum minus* | kollin-subalpin (-alpin) |
| *Thesium alpinum* | montan-alpin |
| *Thesium bavarum* | kollin-montan |
| *Thymus praecox polytrichus* | (colline) subalpine-alpine |
| *Thymus pulegioides* | kollin-subalpin (-alpin) |
| *Thymus serpyllum* | kollin-subalpin (-alpin) |
| *Tofieldia calyculata* | (kollin-) montan-alpin |
| *Tragopogon pratensis* | kollin-montan (-subalpin) |
| *Trifolium aureum* | kollin-montan (-subalpin) |
| *Trifolium badium* | subalpine (-alpine) |
| *Trifolium montanum* | kollin-subalpin (-alpin) |
| *Trifolium pratense* | kollin-subalpin (-alpin) |
| *Trifolium repens* | kollin-subalpin (-alpin) |
| *Trifolium thalii* | subalpine-alpine |
| *Trisetum flavescens* | Kollin-montan (-subalpin) |
| *Trollius europaeus* | (collin-) montane-subalpin (-alpin) |
| *Tussilago farfara* | kollin-subalpin (-alpin) |
| *Urtica dioica* | kollin-subalpin (-alpin) |
| *Vaccinium myrtillus* | kollin-alpin |
| *Vaccinium vitis-idaea* | (Kollin-) montan-subalpin |
| *Valeriana montana* | (montane) subalpine (-alpine) |
| *Valerianella locusta* | kollin-montan |
| *Veratrum album* | montan-alpin |
| *Verbena officinalis* | kollin-montan (-subalpin) |
| *Veronica aphylla* | (montan-) subalpine-alpine |
| *Veronica chamaedrys* | kollin-subalpin (-alpin) |
| *Veronica fruticans* | (montan-) subalpine-alpine |
| *Veronica officinalis* | kollin-subalpin |
| *Veronica persica* | kollin-montan (-subalpin) |
| *Veronica spicata* | kollin-subalpin |
| *Veronica teucrium* | kollin-montan (-subalpin) |
| *Vicia cracca* | kollin-subalpin |
| *Vicia pratensis* | kollin-montan (-subalpin) |
| *Vicia sepium* | kollin-montan (-subalpin) |
| *Vincetoxicum hirundinaria* | kollin-subalpin |
| *Viola calcarata* | subalpine-alpine |
| *Viola hirta* | kollin-montan (-subalpin) |
| *Viola tricolor* | (collinous) montane-subalpine |

**Supplementary File 1f.**

| **Model** | **AICc** | **Marginal R^2^** | **Conditional R^2^** | **RMSE** | **LOOCV RMSE** |
| --- | --- | --- | --- | --- | --- |
| Pace-of-Life | 151.12 | 0.227 | 0.497 | 0.292 | 0.311 |
| All Traits | 220.11 | 0.257 | 0.294 | 0.294 | 0.320 |
| Chlorophyll | 164.57 | 0.163 | 0.422 | 0.294 | 0.313 |
| Leaf Longevity | 161.70 | 0.181 | 0.441 | 0.288 | 0.309 |
| Leaf Nitrogen | 167.70 | 0.161 | 0.402 | 0.292 | 0.313 |
| Leaf Phosphorus | 165.38 | 0.168 | 0.433 | 0.296 | 0.314 |
| Specific Leaf Area | 159.30 | 0.229 | 0.452 | 0.294 | 0.311 |
